# Occurrence of Guillain-Barre Syndrome During the Initial Symptomatic Phase of COVID-19 Disease: Coincidence or Consequence?

**DOI:** 10.7759/cureus.19655

**Published:** 2021-11-17

**Authors:** Rui Seixas, David Campoamor, João Lopes, Teresa Bernardo, Hipólito Nzwalo, Dulce Pascoalinho

**Affiliations:** 1 Department of Internal Medicine, Unidade Local de Saúde do Litoral Alentejano, Santiago do Cacém, PRT; 2 Stroke Unit, Algarve University Hospital Center, Faro, PRT; 3 Faculty of Medicine and Biomedical Sciences, University of Algarve, Faro, PRT; 4 Department of Intensive Care, Unidade Local de Saúde do Litoral Alentejano, Santiago do Cacém, PRT

**Keywords:** viral mimicry, neurotropic virus, viral infections, covid-19, guillain barre syndrome

## Abstract

Viral infections are frequently present before the clinical manifestation of Guillain-Barre syndrome (GBS). Multiple studies on coronaviruses have shown that these viruses have neurotropic characteristics, and their molecular mimicry can induce inflammatory demyelinating neuropathy. Herein, we describe a case of a GBS in an 85-year-old patient infected with SARS-CoV-2, manifested with acute progressive symmetric ascending quadriparesis, urinary dysautonomia, and dysphagia, who responded well to treatment with intravenous human immunoglobulin.

## Introduction

Viral infections are frequently present before the clinical manifestation of Guillain-Barre syndrome (GBS) [1]. Antibodies against SARS-CoV-2 (severe acute respiratory syndrome coronavirus 2) can cross-react with peripheral myelin causing GBS since these viruses have neurotropic characteristics, and their molecular mimicry can induce inflammatory demyelinating neuropathy [2]. Thus, in patients with COVID-19, identification of unexplained neurological manifestations is crucial to early detection and management of GBS. The authors describe a case of an 85-year-old patient infected with SARS-CoV-2, with neurological symptoms of GBS and clinical improvement after treatment with intravenous human immunoglobulin.

## Case presentation

An 85-year-old female, previously independent, with a known medical history of congestive heart failure, atrial fibrillation, arterial hypertension, and no prior vaccination to SARS-CoV-2 or influenza, was admitted to the Emergency Department due to shortness of breath and cough for the past week. She also complained of progressive symmetric and ascending loss of muscle strength in the two days prior to hospitalization. The symptoms progressed from distal limbs to proximal limbs, with greater severity in both arms. She denied any other symptomatology, namely gastrointestinal symptoms. SARS-CoV-2 infection was confirmed by reverse transcription-polymerase chain reaction (RT-PCR) in nasopharyngeal swab sample. With the exception of mild hypoxemia on arterial gasometry, the remaining paraclinical investigation, including chest X-ray, was unremarkable. During the first day of hospitalization, the neurological symptoms worsened, and the patient developed urinary dysautonomia. A lumbar punction was performed, and cerebral spinal fluid (CSF) analysis revealed slight albumin-cytologic dissociation (Table 1). A comprehensive investigation excluded neurotropic viral infection, including SARS-CoV-2 and other infectious agents (Table 1).

**Table 1:** Comprehensive differential diagnosis investigation.

Cerebral spinal fluid - cytochemistry	Result
Leukocytes	1.0 mm^3^ (normal range: 0.0–5.0)
Predominant cells	Lymphocytes
Glucose	93.0 mg/dL (normal range: 40.0–70.0 mg/dL)
Chloride	133.0 mEq/L (normal range: 118.0–132.0 mEq/L)
Microproteins	47.0 mg/dL (normal range: 15.0–45.0 mg/dL)
Cerebral spinal fluid - neurotropic viruses	Result
Herpes simplex (HSV-1 and HSV-2)	Negative
Varicella zoster virus	Negative
Epstein–Barr virus	Negative
Cytomegalovirus	Negative
Human herpesvirus (HHV6 and HHV7)	Negative
Adenoviruses	Negative
Poliovirus	Negative
Enteroviruses	Negative
SARS-CoV-2	Negative
Blood serum	Result
Hepatitis C	Negative
Hepatitis B	Negative
HIV	Negative
Treponema pallidum	Negative
Nasopharyngeal swab	Result
SARS-CoV-2	Positive
Influenza A and B	Negative
Respiratory syncytial virus	Negative
Imaging	Result
Chest X-ray	Normal
Brain tomography	Normal

**Figure 1:**
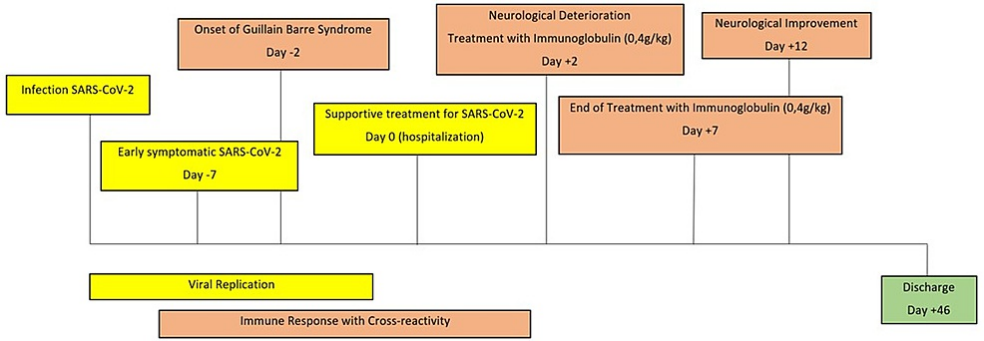
The chronological timeline of the patient's clinical course with essential findings and interventions.

The presence of ascending quadriparesis, dysautonomia, and albumin-cytologic dissociation supported the diagnosis of GBS. Two days after admission, the patient was transferred to a level II intensive care unit and began treatment with intravenous human immunoglobulin with a daily dose of 0.4 gr per kg for five consecutive days (total cumulative dosage of 2 grams per kg) without neurological improvement. The disease progressed with onset of bulbar symptoms, namely dysphagia (Figure 1).

Her hospitalization was prolonged due to aspiration nosocomial pneumonia requiring transient noninvasive ventilation. Finally, after 12 days post-admission, while still needing noninvasive ventilation, her neurological deficits slowly improved with physical rehabilitation, and she was discharged after 46 days of hospitalization, scoring 3 points on the modified Rankin scale. There was no need for corticotherapy.

## Discussion

Infection by SARS-CoV-2 has been shown to lead to multiple neurological manifestations [3], with the most common being dizziness, headache, anosmia, and ageusia [4]. However, some isolated cases of GBS have also been reported worldwide [5]. Rather than a mere coincidence, the occurrence of GBS during early COVID-19 disease should be seen as an example of the SARS-CoV-2 virus’ potential of inducing immune-mediated neurological disease. The incubation period of SARS-CoV-2 infection can be as long as two weeks [6], and viral infections precede GBS's onset in days or weeks [7]. Hence, in this case, the hypothesis of SARS-CoV-2 antibodies cross-reacting with peripheral myelin causing GBS at the beginning of COVID-19 disease is very plausible. Moreover, multiple studies on coronaviruses have shown that these viruses have neurotropic characteristics [8], and molecular mimicry is a mechanism through which SARS-CoV-2 can trigger inflammatory demyelinating neuropathy [2]. Our case highlights the need for prompt identification and clarification of coexistent neurological symptoms such as decreased muscle strength, difficulty in urinating or eating in patients with COVID-19 infection, and adequate management. Even though spinal tap is not indispensable for GBS diagnosis, CSF analysis will help rule out alternative diagnoses. Notably, the presence of SARS-CoV-2 in CSF is usually negative, as reported in several studies, but its positivity has been reported in a single case [9]. Treatment of GBS continues to be intravenous human immunoglobulin even if associated with SARS-CoV-2 infection. Considering the importance of immunomodulation as a treatment of COVID-19 disease, one can speculate if immunoglobulin in our patient contributed to the positive outcome. This question would be better addressed in multicenter databases because of the rarity of concurrent GBS and COVID-19 disease.

## Conclusions

Our case is illustrative of an immune-associated neurological condition induced by SARS-CoV-2 infection. Antibodies against SARS-CoV-2 can cross-react with peripheral myelin causing GBS, and prompt treatment and adequate support to both conditions are essential to increase the chance of a good outcome. Therefore, physicians should be alert that in patients with COVID-19, identification of unexplained neurological manifestations is crucial to early detection and management of neurological pathology.
